# KCNQ1OT1 promotes autophagy by regulating miR‐200a/FOXO3/ATG7 pathway in cerebral ischemic stroke

**DOI:** 10.1111/acel.12940

**Published:** 2019-04-03

**Authors:** Shijia Yu, Mingjun Yu, Xin He, Lulu Wen, Zhongqi Bu, Juan Feng

**Affiliations:** ^1^ Department of Neurology Shengjing Hospital of China Medical University Shenyang China; ^2^ Department of Neurosurgery Shengjing Hospital of China Medical University Shenyang China

**Keywords:** ATG7, autophagy, FOXO3, lncRNA KCNQ1OT1, miR‐200a, stroke

## Abstract

Dysregulation of long noncoding RNAs (lncRNAs) is associated with abnormal development and pathophysiology in the brain. Increasing evidence has indicated that ischemic stroke is becoming the most common cerebral disease in aging populations. The treatment of ischemic stroke is challenging, due in part to ischemia and reperfusion (I/R) injury. In this study, we revealed that potassium voltage‐gated channel subfamily Q member 1 opposite strand 1 (KCNQ1OT1) was significantly upregulated in ischemic stroke. Knockdown of KCNQ1OT1 remarkably reduced the infarct volume and neurological impairments in transient middle cerebral artery occlusion (tMCAO) mice. Mechanistically, KCNQ1OT1 acted as a competing endogenous RNA of miR‐200a to regulate downstream forkhead box O3 (FOXO3) expression, which is a transcriptional regulator of ATG7. Knockdown of KCNQ1OT1 might inhibit I/R‐induced autophagy and increase cell viability via the miR‐200a/FOXO3/ATG7 pathway. This finding offers a potential novel strategy for ischemic stroke therapy.

## 1 INTRODUCTION

Ischemic stroke is considered one of the most serious diseases worldwide, threatening the health and life of aging populations. Patient disabilities after suffering stroke can be significant burdens to families and society as a whole (GBD Mortality & Causes of Death Collaborators, 2015). To date, the most effective therapy for acute ischemic stroke (AIS) is vascular recanalization. However, because of the narrow time window and secondary ischemia and reperfusion (I/R) injury in the penumbra, the efficiency of vascular recanalized therapy is not sufficient (Prabhakaran, Ruff, & Bernstein, 2015; Snow, 2016). Therefore, exploring latent regulatory mechanisms in I/R injury may help overcome these limitations and treatment side effects to some extent.

Noncoding RNAs that are longer than 200 nt are defined as long noncoding RNAs (lncRNAs). LncRNAs participate in several crucial biological processes, such as chromosome remodeling, genomic imprinting, transcriptional activation, post‐transcriptional interference, and intranuclear transport of nucleic acids (Quinn & Chang, 2016). About 40% of the most differentially expressed lncRNAs are brain‐specific (Derrien et al., 2012). LncRNAs are considered to be indispensable in brain development and are likely related to brain impairments (Barry, 2014). Potassium voltage‐gated channel subfamily Q member 1 opposite strand 1 (KCNQ1OT1) is a noncoding antisense RNA that regulates epigenetic silencing as an imprinted gene cluster (Pandey et al., 2008). Increased KCNQ1OT1 levels have been shown to be associated with atherosclerosis and heart attacks, which are high‐risk factors of stroke (Arslan et al., 2017; Vausort, Wagner, & Devaux, 2014). However, the expression and function of KCNQ1OT1 in ischemic stroke are still unknown.

Recent reports suggest that noncoding RNAs can affect cell survival in stroke by regulating autophagy (Guo et al., 2017; Han et al., 2018). Autophagy is a necessary process to degrade impaired organelles and misfolded proteins in eukaryotic cells. It can affect cellular homeostasis during synthesis and catabolism (Kim et al., 2013). Activated autophagy has been detected in numerous diseases, including cancer, AIDs, cardiac‐cerebral vascular and neurodegenerative disorders, and can be either beneficial or detrimental, as a double‐edged sword (Abdellatif, Sedej, Carmona‐Gutierrez, Madeo, & Kroemer, 2018; Grishchuk, Ginet, Truttmann, Clarke, & Puyal, 2011; Zhou & Spector, 2008). Reduction in autophagy has been demonstrated to ameliorate damage caused by focal cerebral infarction (Xing et al., 2012). Autophagy‐related genes are conserved from yeast to mammals. ATG7 encodes an E1‐like ligase, which is essential to form a mature autophagosomal membrane. ATG7 is involved in regulation of cell death (Pattison, Osinska, & Robbins, 2011). As a critical promoter of autophagy, ATG7 is associated with multiple diseases, including cancer, metabolic and neurological diseases (Hu et al., 2018; Martinez‐Lopez & Singh, 2016; Xie et al., 2016). Specific knockout of ATG7 protects against brain injury following hypoxia (Xie et al., 2016). Thus, we are interested in the molecular regulation of ATG7‐dependent autophagy in cerebral I/R injury.

MicroRNAs (miRNAs) are a set of endogenous short noncoding RNAs that can affect mRNA stability, transcription, and translation (Ameres & Zamore, 2013). MiRNAs function by partially or completely combining with the 3'‐UTR of their targeted mRNAs (Liu et al., 2017). Accumulated evidence has shown that lncRNAs can serve as molecular sponges of miRNAs in binding to their downstream genes. For instance, lncRNA MIAT directly combines with miR‐150‐5p and linc00152 is directly targeted by miR‐103a‐3p in a specific‐sequence manner (Yan et al., 2015; Yu et al., 2017). Bioinformatics software (Lncbase) predicts a latent binding site for KCNQ1OT1 and miR‐200a. And we realized that the increase in miR‐200a was greater than others after KCNQ1OT1 knockdown in oxygen and glucose deprivation and re‐oxygenation (OGD/R) cells. MiR‐200a was determined to participate in inflammatory and senescence processes in cells (Zhao et al., 2018). In addition, miR‐200a inhibits proliferation in carcinoma cells (Li et al., 2017). It was reported that miR‐200a regulated autophagy in ovarian cancer via the ATG7 pathway (Hu et al., 2018). MiR‐200a, which is upregulated by thymosin beta 4, could prevent cell death after ischemic injury by regulating the EGFR signaling pathway (Santra et al., 2016). However, the detailed function of miR‐200a in I/R‐induced brain damage needs further exploration.

Forkhead box O3 (FOXO3) was a speculated target of miR‐200a in the miRWalk database. According to previous studies, FOXO3, which belongs to the FOXO family, is a transcription factor that regulates cell autophagy, survival, and senescence in mammals (Accili & Arden, 2004). FOXO3 induces autophagy and inhibits cell proliferation in gastric adenocarcinoma (Gao et al., 2018). A FOXO3‐related pathway is involved in ketone neuroprotection against ischemic stroke (Yin, Han, Tang, Liu, & Shi, 2015). Evidence has shown that increased FOXO3, which is mediated by miRNA‐132 and miRNA‐212, was able to impair cell viability (Wong et al., 2013). Additionally, FOXO3 was identified to direct the autophagy program through regulating multiple downstream genes (Warr et al., 2013). Using the JASPAR database, we found that ATG7 was among the downstream targets of FOXO3. After we confirmed the effect of ATG7‐dependent autophagy in I/R injury, we hypothesized that FOXO3 might function by targeting ATG7.

In the present study, we detected KCNQ1OT1, miR‐200a, FOXO3, and ATG7 expression in tissues and cells after I/R injury. In addition, we explored possible mechanisms that may influence cell viability and autophagy in I/R. We explain the involvement of the KCNQ1OT1/miR‐200a/FOXO3/ATG7 axis in regulating I/R‐induced autophagy. These results may provide a novel understanding of molecular regulation in I/R injury and offer an alternative approach to improve ischemic stroke treatment.

## 2 RESULTS

### 2.1 KCNQ1OT1 was upregulated in focal ischemia

**Figure 1:**
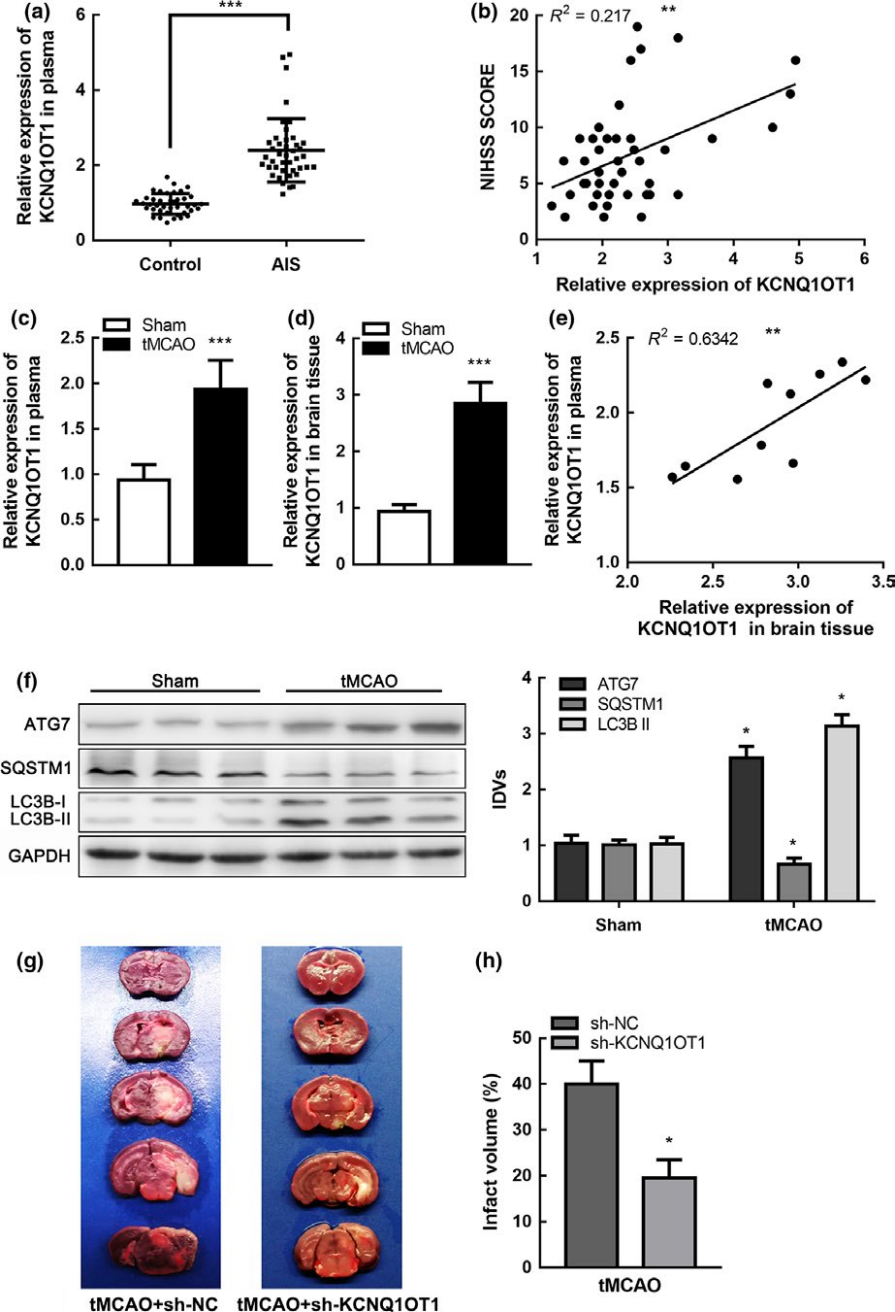
KCNQ1OT1 was upregulated in focal ischemia (a) Expression of KCNQ1OT1 in plasma of AIS patients (*n* = 42) and healthy controls (*n* = 40) detected by real‐time qPCR. Data are presented as the mean ±*SD*. ***p < 0.001 vs. health control group. (b) Linear regression analysis was conducted to each individual about KCNQ1OT1 expression and National Institute of Health Stroke Scale (NIHSS) score, **p < 0.01. (c,d) Expression of KCNQ1OT1 in plasma (c) and brain tissue (d) of tMCAO and sham mice detected by qRT‐PCR. Data are presented as the mean ± *SD* (*n* = 10 in each group). ***p < 0.001 vs. sham group. (e) Linear regression analysis was conducted to each individual KCNQ1OT1 expression in plasma and brain tissue in tMCAO group (*n* = 10), **p < 0.01. (f) Protein levels of ATG7, SQSTM1, and LC3B II in tMCAO and sham mice with GAPDH as an endogenous control. Data are presented as the mean ± *SD* (*n* = 6 in each group). *p < 0.05 vs. sham group. (g) Infarct region was visualized by triphenyltetrazolium chloride (TTC) staining. (h) Infarct size was measured using Image J software. Data are presented as the mean ± *SD* (*n* = 6 in each group). *p < 0.05 vs. tMCAO + sh‐NC group

To determine whether KCNQ1OT1 was related to ischemic stroke, we tested relative expression in AIS patients using Quantitative real‐time PCR (qRT‐PCR). The basic characteristics of participants are listed in Table 1. Compared with controls, KCNQ1OT1 was markedly increased in AIS patients' plasma (Figure 1a). In addition, KCNQ1OT1 expression was positively correlated with stroke severity, which was evaluated based on National Institute of Health Stroke Scale (NIHSS) scores (*R* ^2 ^= 0.2170, *p* < 0.01, Figure 1b). We established the transient middle cerebral artery occlusion (tMCAO) model to simulate I/R in vivo and detected KCNQ1OT1 expression in mice plasma and brain tissue (Figure 1c, d). Statistical analysis revealed that KCNQ1OT1 expression in plasma was positively correlated with expression in brain tissue in tMCAO mice (*R* ^2 ^= 0.6342, *p* < 0.01, Figure 1e). Autophagy was activated in I/R injury, with increased ATG7 expression in mice subjected to tMCAO (Figure 1f).

**Table 1:** Baseline characteristics of the subjects

	AIS (*n* = 42)	Control (*n* = 40)	*p*
Age (year)	64.17 ± 1.336	62.68 ± 1.255	0.419
Male, *n* (%)	22 (52.4)	19 (47.5)	0.659
Smoking, *n* (%)	11 (26.2)	8 (20)	0.507
Drinking, *n* (%)	5 (11.9)	2 (5)	0.263
Hypertension, *n* (%)	31 (73.8)	7 (17.5)	<0.001
Diabetes, *n* (%)	17 (40.5)	13 (32.5)	0.454
Total cholesterol (mmol/L)	4.63 ± 1.18	4.66 ± 0.90	0.228
Triglyceride (mmol/L)	1.54 ± 0.87	1.49 ± 0.94	0.799
LDL‐C (mmol/L)	2.93 ± 0.98	2.94 ± 0.80	0.440
HDL‐C (mmol/L)	1.13 ± 0.39	1.24 ± 0.30	0.415
NIHSS score			
(1–4)	14 (33.33%)		
(5–15)	23 (54.76%)		
(16–25)	5 (11.91%)		

### 2.2 KCNQ1OT1 knockdown alleviated brain injury and inhibited autophagy in tMCAO mice

To investigate the role of KCNQ1OT1 in ischemic stroke in vivo, mice were preinjected with either sh‐KCNQ1OT1 lentivirus or negative control (sh‐NC) lentivirus into their lateral ventricles two weeks before the tMCAO operation. Mice were then sacrificed 24 hr after tMCAO (Supporting Information Figure S1a). Markedly downregulated KCNQ1OT1 expression was observed in the sh‐KCNQ1OT1‐injected mice compared with the sh‐NC‐injected group (Supporting Information Figure S1b). Cerebral infarction volume in mice was stained using 2,3,5‐triphenyltetrazolium chloride (TTC) (Figure 1g). The infarct volume was significantly reduced in the mice injected with sh‐KCNQ1OT1 compared to the sh‐NC group (Figure 1h). Autophagy was inhibited with lower ATG7 expression in tMCAO mice, which were pretreated with sh‐KCNQ1OT1 lentivirus (Supporting Information Figure S1c). To explore the potential influence of KCNQ1OT1 on the development of ischemic stroke, we recorded neurological deficit scores at 1, 3, 7, and 14 days after tMCAO. The data indicated that KCNQ1OT1 knockdown ameliorated neurological impairments in tMCAO mice (Supporting Information Figure S1d). These results suggested that KCNQ1OT1 might participate in the pathogenesis and development of ischemic stroke. In addition, KCNQ1OT1 was likely to regulate autophagy in the tMCAO model.

### 2.3 KCNQ1OT1 knockdown protected cells in OGD/R by inhibiting autophagy

**Figure 2:**
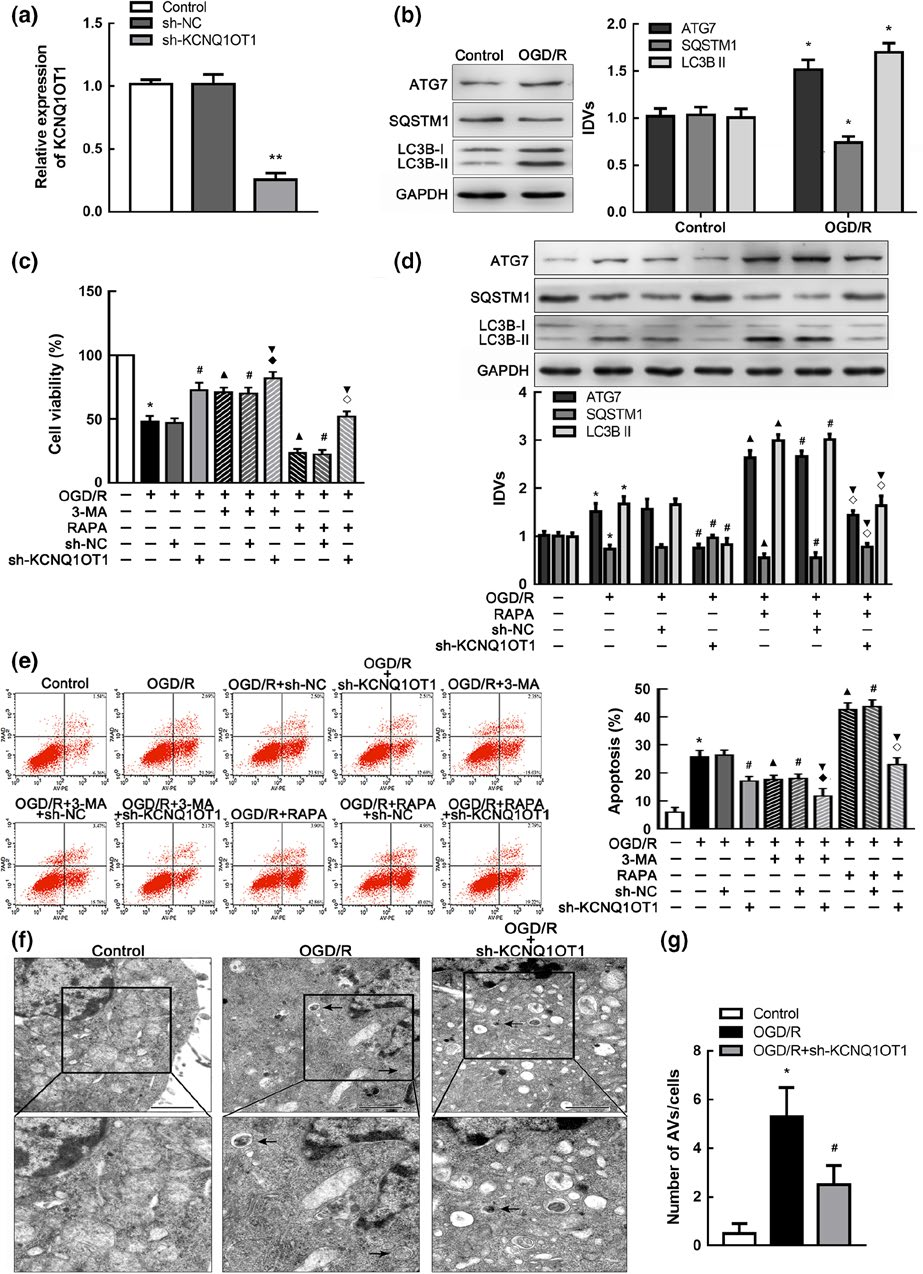
Knockdown of KCNQ1OT1 restrained autophagy in vitro. (a) Expression of KCNQ1OT1 in cells after transfected with sh‐KCNQ1OT1 plasmids and scrambled vectors (NC), respectively. **p < 0.01 vs. sh‐NC group. (b) Western blot analysis of ATG7, SQSTM1, and LC3B II altered expression in OGD/R cells with GAPDH as an endogenous control. *p < 0.05 vs. control group. (c) CCK‐8 assay was performed to assess the influences of KCNQ1OT1, 3‐MA, and rapamycin (RAPA) on cell viability. (d) Western blot analysis of ATG7, SQSTM1, and LC3B II expression in OGD/R cells with the treatment of sh‐KCNQ1OT1 and RAPA. GAPDH was regarded as an endogenous control. (e) Flow cytometry analysis of cells after the intervention of sh‐KCNQ1OT1, 3‐MA, or RAPA. The apoptosis rates equated to the sum percent of right lower quadrant (representing early apoptosis) and right upper quadrant (representing late apoptosis). Statistical analysis was applied with nonparametric Mann–Whitney test. *p < 0.05 vs. control group. ^#^ *p* < 0.05 vs. OGD/R + sh‐NC group. ^▲^ *p* < 0.05 vs. OGD/R group. ^▼^ *p* < 0.05 vs. OGD/R + sh‐KCNQ1OT1 group. ^◆^ *p* < 0.05 vs. OGD/R + 3‐MA + sh‐NC group. ^◇^ *p* < 0.05 vs. OGD/R + RAPA + sh‐NC group. (f) Transmission electron microscopy was applied to observe the ultrastructural features in OGD/R cells with altered KCNQ1OT1 expression. Arrows show autophagic vacuoles (AVs) with double membranes. Scale bars represent 1 µm. (g) The number of AVs was calculated statistically, at least 40 cells were counted in each experiment. *p < 0.05 vs. control group. ^#^ *p* < 0.05 vs. OGD/R group. For (a‐f), data are presented as the mean ± *SD* (*n* = 3 in each group)

Cells were exposed to OGD/R treatment to mimic I/R in vitro. We investigated the regulating mechanism of KCNQ1OT1 in the OGD/R model. We first established the stable sh‐KCNQ1OT1 transfected cell line and verified transfection efficiency (Figure 2a). Autophagy was activated in the OGD/R group via western blot analysis (Figure 2b). To further evaluate the effects of KCNQ1OT1 and autophagy in I/R injury, we applied 3‐methyladenine (3‐MA) as an autophagy inhibitor or rapamycin to induce autophagy. As shown in Figure 2c, impairment of cell viability in OGD/R‐treated cells was alleviated after KCNQ1OT1 knockdown or 3‐MA intervention. This change generated after transfection of sh‐KCNQ1OT1 could be abolished by rapamycin treatment. In addition, autophagy was inhibited in sh‐KCNQ1OT1‐transfected OGD/R cells, while inhibition could be rescued by rapamycin treatment (Figure 2d).

Since apoptosis was identified as crucial programmed cell death, we focused on the impacts of autophagy on OGD/R‐induced apoptosis. We detected the cell apoptosis rate using flow cytometry analysis and terminal‐deoxynucleotidyl transferase‐mediated nick end labeling (TUNEL) assay. The cell apoptosis rate was increased in OGD/R‐treated cells and became lower after treatment with either sh‐KCNQ1OT1 plasmids or 3‐MA. Rapamycin intervention negated the reduction in apoptosis rates due to KCNQ1OT1 knockdown (Figure 2e; Supporting Information Figure S2a).

To further explore the activation of autophagy, we immunostained the autophagosome marker LC3B and found that transfection with sh‐KNQ1OT1 markedly suppressed OGD/R‐induced autophagy (Supporting Information Figure S2b). In addition, autophagic vacuoles (AVs) were observed by transmission electron microscopy (TEM), which were characterized as sequestration of organelles or portions of the cytoplasm with double membranes (Klionsky et al., 2016). Counting showed that the increased number of AVs in OGD/R cells could be inhibited after transfection with sh‐KCNQ1OT1 (Figure 2f). Moreover, autophagy flux was monitored by mRFP‐GFP‐LC3B adenovirus infection, which traced LC3B through red or green fluorescent distribution. Green fluorescent protein (GFP) was labile in an acidic environment, for example, in autolysosomes where the red fluorescent protein (RFP) was stable. Colocalization of GFPs and RFPs was observed in early autophagosomes, which appeared as yellow puncta. When autolysosomes were formed with the fusion of autophagosomes and lysosomes, pH value in cells fell below 5, and the GFPs were subsequently degraded with only the RFPs left (Wang et al., 2014). The fluorescent density and intensity of yellow and red puncta were greatly increased after OGD/R treatment, whereas fewer yellow and red puncta occurred in the sh‐KCNQ1OT1‐transfected group (Supporting Information Figure S2c).

These results suggested that activated autophagy could promote cell apoptosis in I/R injury. KCNQ1OT1 knockdown might protect cells by mediating I/R‐induced autophagy.

### 2.4 miR‐200a promoted cell survival in OGD/R

**Figure 3:**
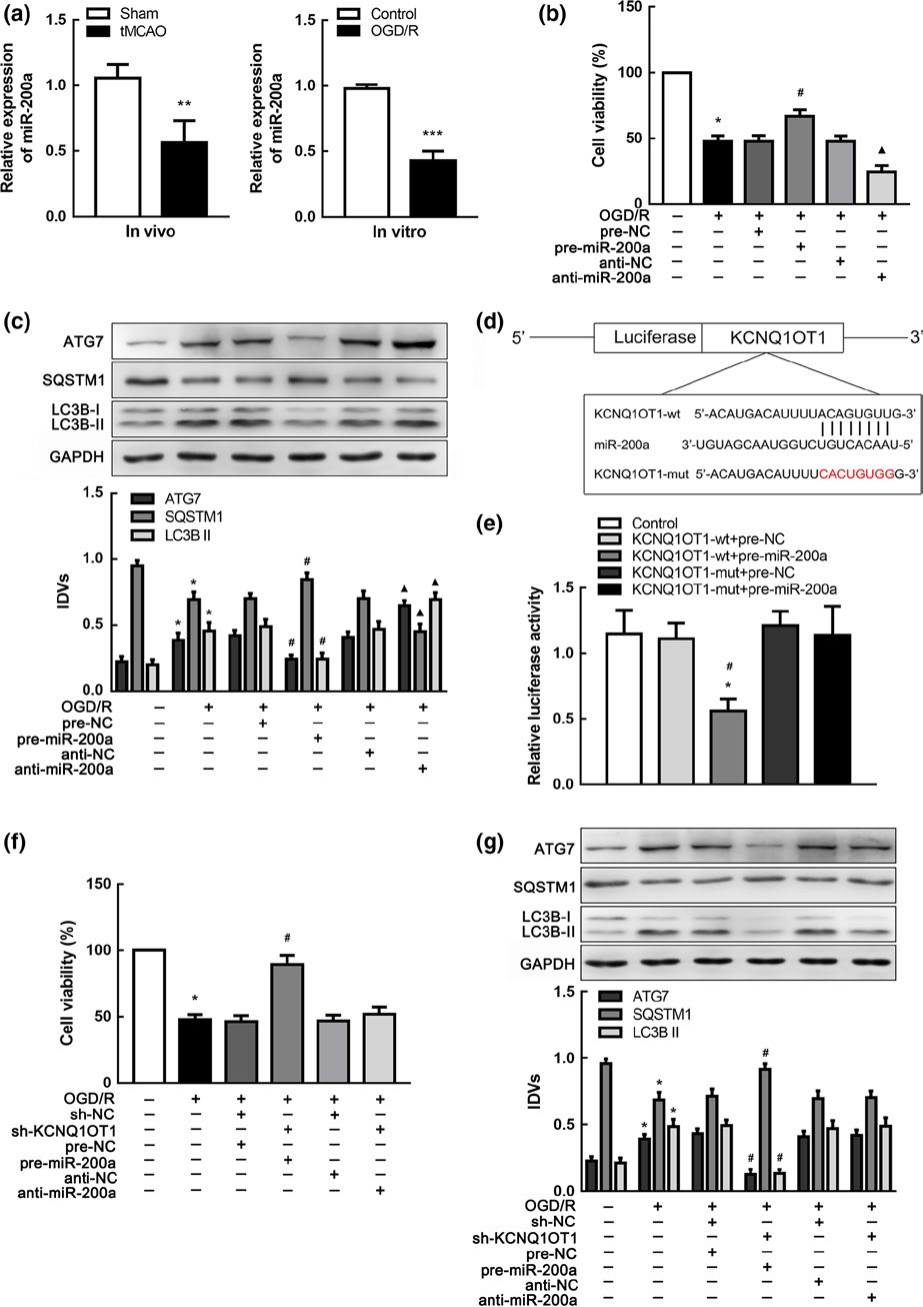
miR‐200a promoted cells survival and was targeted by KCNQ1OT1 in OGD/R‐induced autophagy. (a) qRT‐PCR analysis of miR‐200a expression in tMCAO (left panel, *n* = 10) and OGD/R cells (right panel, *n* = 5). Data are presented as the mean ± *SD*. **p < 0.01 vs. sham group. ***p < 0.001 vs. control group. (b) CCK‐8 assay was conducted to explore the influence of changing miR‐200a on cell viability in OGD/R. (c) Western blot analysis of ATG7, SQSTM1, and LC3B II with altered miR‐200a using GAPDH as an endogenous control. *p < 0.05 vs. control group. ^#^ *p* < 0.05 vs. OGD/R + pre‐NC group. ^▲^ *p* < 0.05 vs. OGD/R + anti‐NC group. (d) Presentation of the putative binding site of miR‐200a and KCNQ1OT1 (KCNQ1OT1‐wt), and the designed mutant sequence (KCNQ1OT1‐mut). (e) The relative luciferase activities of N2a cells co‐transfected either KCNQ1OT1‐wt or KCNQ1OT1‐mut with either pre‐miR‐200a or pre‐NC. *p < 0.05 vs. KCNQ1OT1‐wt + pre‐NC. ^#^ *p* < 0.05 vs. KCNQ1OT1‐mut + pre‐miR‐200a. (f) CCK‐8 assay was performed to evaluate the effects of KCNQ1OT1 and miR‐200a on cell viability. (g) Western blot analysis of ATG7, SQSTM1, and LC3B II affected by KCNQ1OT1 accompanied with miR‐200a alteration using GAPDH as an endogenous control. *p < 0.05 vs. control group. ^#^ *p* < 0.05 vs. OGD/R + sh‐NC + pre‐NC group. For (b,e,f,g), data are presented as the mean ± *SD* (*n* = 3 in each group)

To further profile the molecular mechanism of KCNQ1OT1 regulating autophagy in I/R injury, we explored miRNA expression to determine miR‐200a as the most affected downstream molecular target using miRNA microarray (Supporting Information Figure S3a). MiR‐200a had been previously proven to be conducive to cell survival in ischemia (Santra et al., 2016). In this study, miR‐200a was downregulated after I/R injury both in vivo and in vitro (Figure 3a). Cells were then transfected with either overexpressed or silenced miR‐200a plasmids for further investigation. The viability of cells transfected with miR‐200a agomir (pre‐miR‐200a) was significantly improved after OGD/R compared with the negative control (pre‐NC) group (Figure 3b). ATG7 expression was decreased, and autophagy was repressed in OGD/R cells transfected with pre‐miR‐200a (Figure 3c). Hence, miR‐200a may be related to regulation of cell viability and autophagy in I/R injury.

### 2.5 KCNQ1OT1 knockdown inhibited autophagy by upregulating miR‐200a

Data indicated a negative correlation between KCNQ1OT1 and miR‐200a expression in the brain tissue of tMCAO mice (*R* ^2 ^= 0.6875, *p* < 0.01, Supporting Information Figure S3c). To assess KCNQ1OT1 regulation of miR‐200a in I/R injury, we detected miR‐200a expression in sh‐KCNQ1OT1 cells following OGD/R and showed that miR‐200a expression was increased in cells transfected with sh‐KCNQ1OT1 (Supporting Information Figure S3d). In contrast, cells transfected with pre‐miR‐200a expressed lower KCNQ1OT1 levels in OGD/R (Supporting Information Figure S3e). Searching the bioinformatics database (Lncbase), we speculated a putative binding site between KCNQ1OT1 and miR‐200a (Figure 3d), which was confirmed by dual‐luciferase gene reporter assay. Relative luciferase activity was significantly weakened in the KCNQ1OT1‐wt + pre‐miR‐200a group (Figure 3e), suggesting that KCNQ1OT1 is a direct target of miR‐200a. Next, sh‐KCNQ1OT1 was co‐transfected with pre‐miR‐200a or anti‐miR‐200a to investigate regulation in I/R injury. Cell viability was improved in the sh‐KCNQ1OT1 + pre‐miR‐200a group compared with the sh‐NC + pre‐NC group (Figure 3f). In addition, ATG7 and LC3B II protein levels were significantly reduced in the sh‐KCNQ1OT1 +pre‐miR‐200a group, indicating that autophagy was inhibited (Figure 3g). Increased cell viability and suppression of autophagy were abolished when transfected with anti‐miR‐200a, implying that KCNQ1OT1 knockdown might support cell survival and repress autophagy of OGD/R cells by binding with miR‐200a.

### 2.6 FOXO3 was upregulated and induced autophagy in OGD/R

**Figure 4:**
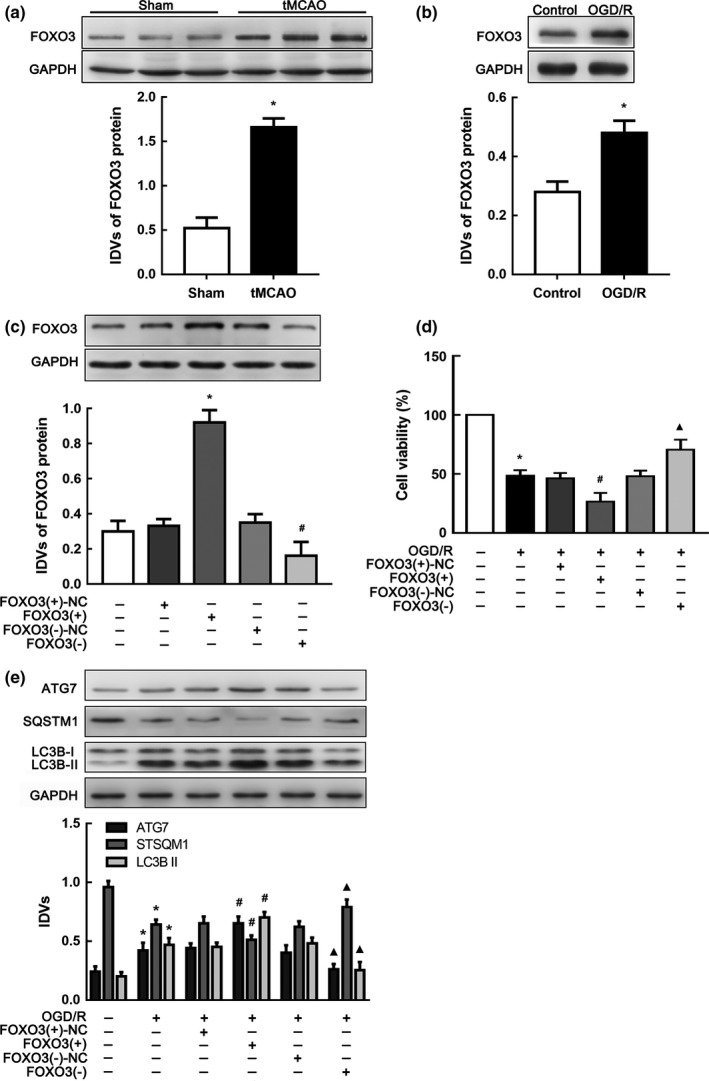
FOXO3 was upregulated and attenuated cell viability in ischemia and reperfusion (I/R). (a) Western blot analysis of FOXO3 in brain tissue of tMCAO mice (*n* = 6 in each group). *p < 0.05 vs. sham group. (b) Western blot analysis of FOXO3 in OGD/R cells. *p < 0.05 vs. control group. (c) Altered protein levels of FOXO3 after transfection of FOXO3(+), FOXO3(−) plasmids or their scrambled vectors (NC), respectively. *p < 0.05 vs. FOXO3(+)‐NC group. ^#^ *p* < 0.05 vs. FOXO3(−)‐NC group. (d) CCK‐8 assay was applied to estimate the impacts of FOXO3 expressing alteration on OGD/R cell viability. (e) Western blot analysis of ATG7, LC3B II and SQSTM1 expression influenced by changing FOXO3 protein levels with GAPDH as an endogenous control. *p < 0.05 vs. control group. ^#^ *p* < 0.05 vs. OGD/R + FOXO3(+)‐NC group. ^▲^ *p* < 0.05 vs. OGD/R + FOXO3(−)‐NC. For (b–e), data are presented as the mean ± *SD* (*n* = 3 in each group)

FOXO3 has been reported to be associated with ischemic stroke (Yin et al., 2015) and may participate in regulation of autophagy (Warr et al., 2013). To verify the role of FOXO3 in I/R injury, we detected FOXO3 protein levels both in brain tissues and cells. As shown in Figure 4a, b, FOXO3 protein expression was apparently increased after I/R. To further investigate, we established stable transfected FOXO3 overexpressing or knockdown cell strains and determined transfection efficiency according to FOXO3 protein expression (Figure 4c). FOXO3‐overexpressed cells had reduced viability after OGD/R compared with those in the negative control group (Figure 4d). Moreover, ATG7 and LC3B II protein expression was upregulated in FOXO3(+) cells compared with FOXO3(+)‐NC (nontargeting sequences of FOXO3(+) plasmid) cells subjected to OGD/R (Figure 4e, f). These results indicated that FOXO3 reduced cell viability and activated autophagy in I/R injury.

### 2.7 miR‐200a targeted FOXO3 3'‐UTR and inhibited its expression

**Figure 5:**
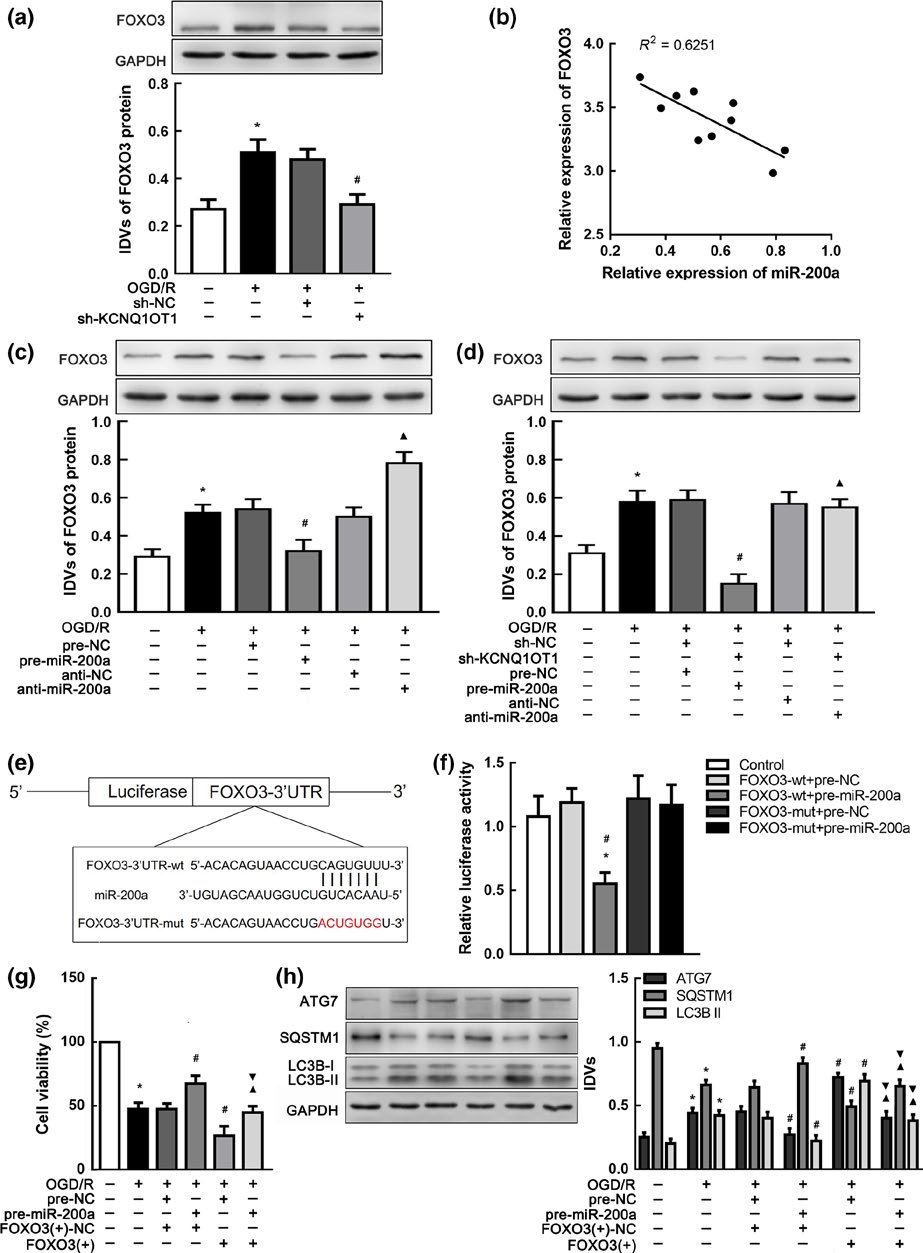
FOXO3 was a target gene of miR‐200a and modulated by both KCNQ1OT1 and miR‐200a. (a) Western blot analysis of FOXO3 affected by KCNQ1OT1 knockdown in OGD/R. *p < 0.05 vs. control group. ^#^ *p* < 0.05 vs. OGD/R + sh‐NC group. (b) Linear regression analysis was conducted to each individual expression of miR‐200a and FOXO3 in tMCAO mice (*n* = 10). **p < 0.01. (c) Western blot analysis of FOXO3 expression affected by altered miR‐200a. *p < 0.05 vs. control group. ^#^ *p* < 0.05 vs. OGD/R + pre‐NC group. ^▲^ *p* < 0.05 vs. OGD/R + anti‐NC group. (d) Western blot analysis of FOXO3 expression affected by altered KCNQ1OT1 and miR‐200a with GAPDH as an endogenous control. *p < 0.05 vs. control group. ^#^ *p* < 0.05 vs. OGD/R + sh‐NC + pre‐NC group. (e) Presentation of the putative binding site of miR‐200a and FOXO3 (FOXO3‐wt), and the designed mutant sequence (FOXO3‐mut). (f)The relative luciferase activities of N2a cells co‐transfected FOXO3‐wt or FOXO3‐mut with pre‐miR‐200a or pre‐NC. *p < 0.05 vs. FOXO3‐wt + pre‐NC. ^#^ *p* < 0.05 vs. FOXO3‐mut + pre‐miR‐200a. (g) CCK‐8 assay was used to investigate the effect of altered miR‐200a and FOXO3 expression on OGD/R cells viability. (h) Western blot analysis of ATG7, SQSTM1, and LC3B II expression impacted by altered miR‐200a and FOXO3. *p < 0.05 vs. control group. ^#^ *p* < 0.05 vs. OGD/R + pre‐NC + FOXO3(+)‐NC group. ^▲^ *p* < 0.05 vs. OGD/R + pre‐miR‐200a + FOXO3(+)‐NC group. ^▼^ *p* < 0.05 vs. OGD/R + pre‐NC + FOXO3(+) group. For (a,c,d,f–h), data are presented as the mean ± *SD* (*n* = 3 in each group).

Given that FOXO3 was confirmed as an autophagy motivator in I/R injury, we hypothesized that FOXO3 may be involved in autophagy modulated by KCNQ1OT1. First, we recognized that FOXO3 expression was decreased in OGD/R cells after KCNQ1OT1 knockdown (Figure 5a). Since FOXO3 expression was negatively correlated with miR‐200a in the brain of tMCAO mice (Figure 5b), we wondered whether altered miR‐200a might affect FOXO3 expression. As shown in Figure 5c, FOXO3 expression was increased in the anti‐miR‐200a group subjected to OGD/R. Furthermore, we verified that reduction in FOXO3 expression after sh‐KCNQ1OT1 transfection could be counteracted by transfection of anti‐miR‐200a in OGD/R cells (Figure 5d). Bioinformatics database (miRWalk) provided a putative target site between miR‐200a and FOXO3 3'UTR (Figure 5e), and a dual‐luciferase activity assay was performed to confirm FOXO3 3'‐UTR as a direct downstream target of miR‐200a. The cells, which were transfected with both FOXO3‐wt and pre‐miR‐200a (FOXO3‐wt + pre‐miR‐200a), showed greatly impaired relative luciferase activity compared with other groups (Figure 5f). As shown in Figure 5g, increased cell viability induced by pre‐miR‐200a transfection was negated by co‐transfection with FOXO3 (+) in the OGD/R group. Autophagy was activated with increasing ATG7 expression in the pre‐miR‐200a transfected group, while upregulation of FOXO3 counteracted the effect (Figure 5h). These results supported that FOXO3 was a downstream target of the KCNQ1OT1/miR‐200a pathway in I/R injury.

### 2.8 ATG7 was involved in FOXO3‐mediated autophagy in OGD/R

**Figure 6:**
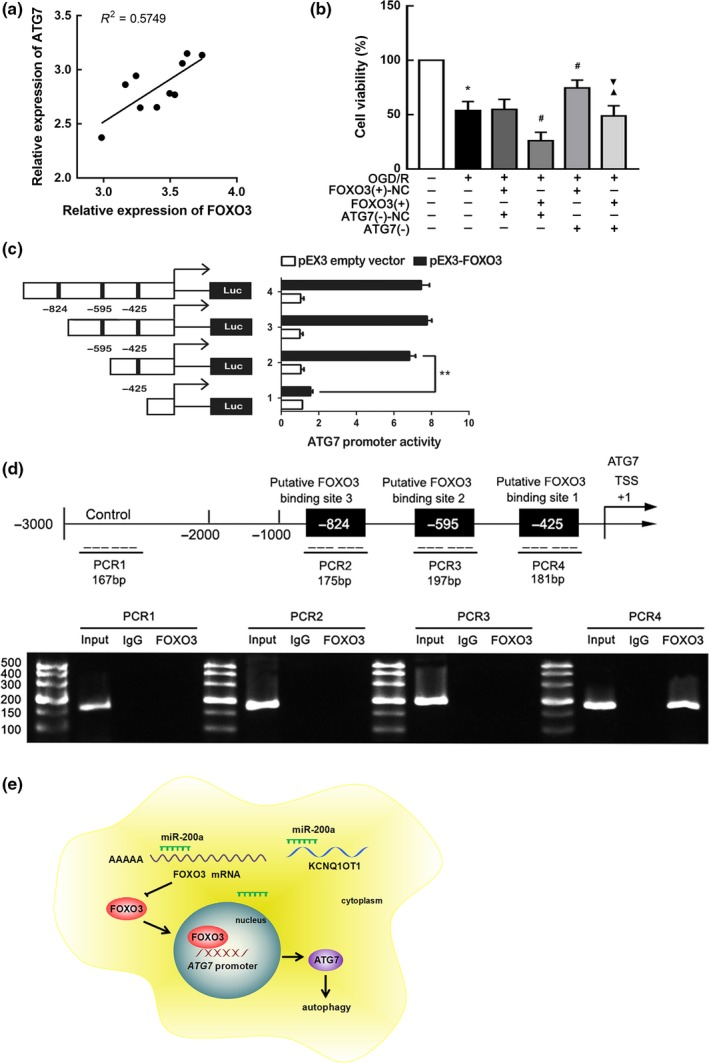
ATG7 was a downstream gene of KCNQ1OT1/miR‐200a/FOXO3 axis. (a) Linear regression analysis was conducted to each individual expression of FOXO3 and ATG7 in tMCAO mice (*n* = 10). **p < 0.01. (b) CCK‐8 assay was conducted to investigate the effect of altered FOXO3 and ATG7 expression on OGD/R cells viability. Data are presented as the mean ± *SD* (*n* = 3 in each group). *p < 0.05 vs. control group. ^#^ *p* < 0.05 vs. OGD/R + FOXO3(+)‐NC + ATG7(−)‐NC group. ^▲^ *p* < 0.05 vs. OGD/R + FOXO3(+) + ATG7(−)‐NC group. ^▼^ *p* < 0.05 vs. OGD/R + FOXO3(+)‐NC + ATG7(−) group. (c) Different reporter constructs with schematic depiction was applied, and the luciferase activity is detected. The Y‐bar shows the deletions on the DNA fragments. X‐bar shows the plasmid activity with normalized to the co‐transfection of reference vector, and relative activity to pEX3 empty vector, which activity was set to 1. Data are presented as the mean ± *SD* (*n* = 3, each). (d) Presentation of the predicted binding site for FOXO3 and ATG7 promoter region 3,000 bp upstream of the transcription start site (TSS) which designated as +1. Immunoprecipitated DNA was amplified by PCR. Normal rabbit IgG was used as a negative control. (e) The schematic description of the mechanism of KCNQ1OT1/miR‐200a/FOXO3/ATG7 axis in regulating autophagy during ischemia and reperfusion (I/R) injury

ATG7 is known as an E1‐like ligase, which is essential in autophagy processes (Mizushima & Komatsu, 2011; Nishida et al., 2009; Pattison et al., 2011). Knockout of ATG7 could promote cell survival during hypoxia (Xie et al., 2016). ATG7 was predicted to be directly targeted by FOXO3 in the JASPAR database. The present study found a positive correlation between FOXO3 and ATG7 in the brain of mice subjected to tMCAO (Figure 6a). As shown in Figure 4e, ATG7 expression was increased in the FOXO3 (+) group. To detect detailed mechanism regarding FOXO3 regulation of ATG7, cell lines with stable transfection of ATG7 knockdown were established (Supporting Information Figure S4a). As shown in Supporting Information Figure S4b, cell viability was enhanced in ATG7 (−)‐transfected cells. Co‐transfection with FOXO3 (+) abolished this effect (Figure 6b). Thus, FOXO3 might affect cell viability in I/R injury via an ATG7‐dependent pathway.

To investigate whether FOXO3 might bind with the promoter of ATG7, luciferase assays were performed. The promoter sequence of ATG7 was determined by searching the Ensembl Genomes database. Three putative FOXO3 binding sites were determined by analyzing DNA sequences in the 1,000 bp upstream region of the transcription start site (TSS) and its 100 bp downstream sequence. Wild‐type deletion constructs and putative FOXO3 binding sites are shown in Figure 6c. Deletion of the −425 site region significantly inhibited the promoter activity of ATG7 after co‐transfection with pEX3‐FOXO3 (Figure 6c). These results indicated that the necessary element affecting an increase in ATG7 promoter activity was likely located in the −425 site region.

To further verify whether FOXO3 directly targeted the promoter of ATG7, chromatin immunoprecipitation (ChIP) assays were conducted. For a negative control, PCR was applied to amplify the 2,000 bp upstream region of the putative FOXO3 binding site, which was not considered to be associated with FOXO3. Immunoprecipitation results illustrate a direct association between FOXO3 and the ATG7 promoter at putative binding site 1 (Figure 6d). However, there were no associations between FOXO3 and other putative ATG7 binding sites and control regions. This finding indicated that FOXO3 likely modulated ATG7 at the transcriptional level.

## 3 DISCUSSION

In this study, we confirmed for the first time that KCNQ1OT1 is highly expressed in AIS patient plasma and can be used to indicate the severity of ischemic stroke. In vivo experiments revealed that KCNQ1OT1 knockdown hindered cerebral infarction and neurological deficits. Furthermore, KCNQ1OT1 knockdown improved cell viability and apoptosis by modulating autophagy in an OGD/R model. Mechanistically, KCNQ1OT1 might target miR‐200a and negatively regulate its expression. KCNQ1OT1 knockdown inhibited autophagy and enhanced cell viability in OGD/R. MiR‐200a might act as a neural protector by restraining autophagy. However, FOXO3 likely hindered the neuroprotective effects of miR‐200a by directly binding with its 3'‐UTR. ATG7 was essential in autophagy activation. Knockdown of ATG7 could negate the influence of FOXO3 on cell viability in OGD/R, and FOXO3 targeted ATG7 promoter directly. Hence, KCNQ1OT1 knockdown might inhibit I/R‐induced autophagy through miR‐200a/FOXO3/ATG7 pathway (Figure 6e).

Ischemic stroke is difficult to treat due to the complex processes involved in I/R injury, including apoptosis and autophagy. Autophagy has been proven to be a continuous physiological process and likely to aggravate apoptosis through excessive degradation (Mizushima, Yoshimori, & Levine, 2010). Previous reports have already confirmed that the activation of autophagy exacerbates cortical neuron death via the Beclin1 pathway (Fan et al., 2016; Grishchuk et al., 2011). Similar to these results, our findings verified that ATG7‐dependent autophagy might damage cell viability in I/R injury. Early evidence has demonstrated that KCNQ1OT1 is associated with risk factors for ischemic stroke, for example, diabetes and myocardial infarction (Vausort et al., 2014). Thus, we hypothesized that KCNQ1OT1 might be dysregulated and exert crucial functions in ischemic stroke. Cells treated with 3‐MA or sh‐KCNQ1OT1 showed an increase in cell viability and a decrease in the apoptosis rate compared with the OGD/R group. However, the advantages of sh‐KCNQ1OT1 were negated by rapamycin, indicating that KCNQ1OT1 knockdown protected OGD/R cells through suppressing autophagy. However, the complicated mechanisms of KCNQ1OT1 in regulating cell viability will require further exploration.

To investigate the detailed molecular regulation of KCNQ1OT1 on autophagy, we detected downstream miRNAs expression after sh‐KCNQ1OT1 transfection. And we found that miR‐200a was the most upregulated miRNA. Thus, we furtherly focused on miR‐200a in the regulation of KCNQ1OT1. According to early research, miR‐200a is involved in tumor, brain injury, cardiomyopathy, and diabetic nephropathy (Fang et al., 2015; Na et al., 2018; Osei et al., 2018; Wei et al., 2014). In addition, miR‐200a ameliorates cell impairment through ischemic preconditioning (Lee et al., 2010). Upregulation of miR‐200a mediates cell protection in cerebral ischemia (Santra et al., 2016). In this study, we found that miR‐200a was downregulated in I/R injury, while KCNQ1OT1 was upregulated. Overexpression of miR‐200a inhibited autophagy and elevated cell viability. MiR‐200a knockdown could abolish the inhibition of autophagy after transfected with sh‐KCNQ1OT1. Our study revealed that miR‐200a was a possible competing endogenous RNAs (ceRNAs) of KCNQ1OT1, which regulated its expressing and functioning (Thomson & Dinger, 2016). Although we considered KCNQ1OT1 as a regulator of miR‐200a in OGD/R‐induced autophagy activation, other lncRNA or molecules were not excluded as possibly being involved in miR‐200a regulation.

Mounting evidence has indicated that miRNAs could mediate downstream gene expression by binding to their 3'‐UTR regions (Ameres & Zamore, 2013). The miRWalk database predicted that miR‐200a might target FOXO3 3'‐UTR. FOXO expression is progressively increased in the aging brain to prevent axonal degeneration (Hwang et al., 2018). FOXO3 was confirmed to promote autophagy via various pathways (Warr et al., 2013; Zhou et al., 2012). Given that FOXO3 could induce autophagy in I/R injury and bind with miR‐200a, we hypothesized that FOXO3 regulation of autophagy is mediated by miR‐200a. In the present study, autophagy induced after FOXO3 overexpression could be counteracted by pre‐miR‐200a. This result implied that miR‐200a might regulate autophagy via FOXO3 pathway. However, the modulating mechanism in organism is a network and miR‐200a might regulate autophagy by other targets. According to the previous study, miR‐200a could mediate ATG7 in ovarian cancer (Hu et al., 2018). Our finding suggests a possible novel mechanism of miR‐200a in regulating I/R‐induced autophagy.

Previous evidence has implied that FOXO3 is associated with multiple genes during autophagy as a transcription factor (Warr et al., 2013). We tested the expression of several FOXO3 targets in OGD/R cells to find that ATG7 and LC3B expressing changed most obviously (Supporting Information Figure S5). In addition, CHIP assay confirmed ATG7 as a direct downstream gene of FOXO3. It was reported that myocardial infarction could induce increased ATG7 expression (Wang et al., 2015). Consistent with this finding, the results of the present study indicate that ATG7 is upregulated in I/R injury of the brain. ATG7 exerted crucial effects on autophagy, such as conversion from LC3B I to LC3B II and autophagic vacuole transport in the cytoplasm (Nishida et al., 2009). Early research found that ATG7‐dependent autophagy exacerbated cortical neuron death (Grishchuk et al., 2011). In this study, we revealed that FOXO3 might aggravate autophagy in I/R injury by directly promoting ATG7 expression as a transcription factor. FOXO3 is also likely to regulate autophagy by targeting other molecules or through other pathways in I/R injury of brain, which will require more exploration in the future.

In conclusion, these results revealed KCNQ1OT1 upregulation in I/R injury of the brain and underlying molecular mechanisms. This study illustrated the involvement of the KCNQ1OT1/miR‐200a/FOXO3/ATG7 axis in regulating autophagy induced by I/R injury. These findings might provide a novel strategy in the treatment of ischemic stroke.

## 4 EXPERIMENTAL PROCEDURES

### 4.1 Human peripheral blood sample collection

A total of 42 patients who suffered AIS for the first time were recruited in the Neurology Department of Shengjing Hospital from October 2016 to July 2017. The diagnoses were confirmed in accordance with clinical manifestations and physical signs using either CT or MRI scans. The degree of neurological impairment was estimated based on NIHSS by two experienced neurologists. Patients with cerebral hemorrhage, tumor, cardiovascular disease, diabetes, or severe systemic diseases were excluded. Blood samples were collected within three hours after the onset of AIS. A total of 40 healthy donors were enrolled from a medical examination center at Shengjing Hospital, China Medical University. All the participants or their legal representatives signed written informed consent for the study.

### 4.2 Animals

All experiments related to animals were approved by the Institutional Animal Care and Use Committee of China Medical University. This study was conducted in complete compliance with the National Institutes of Health Guide for the Care and Use of Laboratory Animals. We spared no effort to minimize the stress and pain of animals used in this trial. Male C57BL/6J mice weighing 22 to 25 g (8–10 weeks old) were provided by Beijing HFK Bioscience Cooperation, China. Mice were maintained in the standard environment of 22°C and 70% humidity in a 12 hr light/dark cycle, with access to food and water ad libitum. Lentivirus encoding short‐hairpin RNA targeting KCNQ1OT1 and its nontargeting sequence were used to infect mice brain tissue. Mice were divided into four groups (*n* = 10 per group): sham, tMCAO, tMCAO + sh‐NC, and tMCAO + sh‐KCNQ1OT1. LV3‐CMV‐GFPPuro‐sh‐KCNQ1OT1 or LV3‐CMV‐GFPPuro‐sh‐NC (5 µl of 10^8^ TU/ml; GenePharma, Shanghai, China) was stereotaxically injected into the lateral ventricle via a Hamilton microsyringe (Hamilton Co., Reno, NV, USA) two weeks before tMCAO. The injection points were located anteroposterior (AP) −0.3 mm, mediolateral (ML) 1.00 mm, and dorsoventral (DV) −2.2 mm to the bregma.

### 4.3 Transient middle cerebral artery occlusion

A tMCAO mouse model was used to establish transient focal cerebral ischemia in vivo on the basis of previous studies. After fasting for eight hours, anesthesia was induced in an acrylic chamber of 3% isoflurane mixed with 70% nitrogen and 30% oxygen and maintained with 1.5% isoflurane through a facemask. The surgery was performed under a stereomicroscope. Left carotid arteries were exposed through a longitudinal median neck incision. The common carotid artery (CCA) was blocked temporarily using artery clamps, and the external carotid artery (ECA) was distally incised with 6‐0 suture ligation. A 6‐0 silicone rubber‐coated nylon monofilament (Beijing Cinontech Co. Ltd, Beijing, China) was inserted into the ECA from the incision and advanced 9–10 mm via the internal carotid artery (ICA) until the origin of the middle cerebral artery (MCA). After 60 min of occlusion, the monofilament was withdrawn and the clamp on CCA was removed for reperfusion. Sham mice underwent an identical procedure without occlusion as controls. During the operation, rectal temperature was controlled at 37 ± 0.5°C using a heating pad.

### 4.4 Neurological deficit assessment

Neurological deficits were determined using modified neurological severity scores (mNSS), which ranged from 0 to 10 (1 to 4, mild; 5 to 9, moderate; 10 to 14, severe injury). The mNSS were recorded at 1, 3, 7, and 14 days after tMCAO by investigators blinded to experimental groups.

### 4.5 Infarct volume measurement

Mice were sacrificed at 24 hr after tMCAO. Brains were cut into 2‐mm‐thick slices and stained with 2% 2,3,5‐triphenyltetrazolium chloride (TTC; Sigma, St. Louis, MO, USA) at 37°C for ten minutes. The infarct volume (%) was computed by Image J software (Bethesda, MD, USA) with the formula: [(volume of the contralateral hemisphere‐volume of the nonischemic ipsilateral hemisphere)/volume of the contralateral hemisphere] × 100.

### 4.6 Cell culture

Mouse neuroblastoma Neuro‐2a (N2a) cells were purchased from the Cell Bank of the Chinese Academy of Sciences (Shanghai, China) and cultured in Dulbecco's modified Eagle medium (DMEM)/high glucose with 10% (v/v) fetal bovine serum (FBS; Gibco, Carlsbad, CA, USA). Cells were grown in a humidified environment of 5% CO_2_ at 37°C.

### 4.7 Oxygen and glucose deprivation and re‐oxygenation and drug intervention in cells

To mimic ischemic/reperfusion (I/R) in cells, an OGD/R model was applied. Cell medium was replaced with glucose‐free DMEM (Gibco, Carlsbad, CA, USA) in an anaerobic incubator for 6 hr after washing three times. The medium had been pretreated by bubbling for 30 min. Cells were placed into normal medium with normoxia for another 24 hr. Drug intervention was performed using either 1 mM 3‐methyladenine (3‐MA; Sigma‐Aldrich, St. Louis, MO, USA) to inhibit autophagy or 200 nM rapamycin (RAPA; Sigma‐Aldrich, St. Louis, MO, USA) to activate autophagy.

### 4.8 Quantitative real‐time PCR

Total RNA was extracted from plasma, brain tissue, and cells using TRIzol reagent (Life Technologies Corporation, Carlsbad, CA, USA). RNA concentrations were detected, and the quality was determined at 260/280 nm absorbance using an Ultra‐micro UV Spectrophotometer (N50 Touch; Implen, Germany). One Step SYBR PrimeScript RT‐PCR Kit (Takara Bio, Inc., Japan) was applied to determine KQNQ1OT1 expression. TaqMan MicroRNA Reverse Transcription Kit (Applied Biosystems, Foster City, CA, USA) was used to reverse transcribe cDNA from miRNA. Quantification reactions were implemented using TaqMan Universal Master Mix II with TaqMan microRNA assays for mmu‐miR‐200a and U6 (Applied Biosystems, Foster City, CA, USA) via the ABI 7,500 Fast Real‐Time PCR System (Applied Biosystems, Foster City, CA, USA). The fold change was equivalent to the relative expression normalized to endogenous control (2^−ΔΔCt^). The primers sequences were as follows: hsa‐KCNQ1OT1: forward 5'‐TGCAGAAGACAGGACACTGG‐3' and reverse 5'‐CTTTGGTGGGAAAGGACAGA‐3'; hsa‐GAPDH: forward 5'‐ TGCACCACCAACTGCTTAGC‐3' and reverse 5'‐GGCATGCACTGTGGTCATGAG‐3'; mmu‐KCNQ1OT1: forward 5'‐GCCAAGCCTGATGACCTAAACTC‐3' and reverse 5'‐ ACTCCCCATCCTCAATTCCTCTA‐3'; and mmu‐GAPDH: forward 5'‐ CCTCGTCCCGTAGACAAAATG‐3' and reverse 5'‐TGAGGTCAATGAAGGGGTCGT‐3'.

### 4.9 Western blot

Tissues and cells were kept in ice‐cold RIPA buffer with protease (Sigma, St. Louis, MO, USA) inhibitor to extract total proteins at 24 hr after treatment. Proteins were separated on a 12% SDS‐PAGE and electrophoretically transferred to polyvinylidene difluoride (PVDF) membranes (Millipore, Billerica, MA). Membranes were then blocked in 5% nonfat milk with Tris‐buffered saline and 0.1% Tween 20 at room temperature for 2 hr. Primary antibodies were incubated at 4°C for at least 12 hr, including LC3B (1:1,000, Cell Signaling Technology, Beverly, MA, USA), SQSTM1 (1:1,000, Cell Signaling Technology, Beverly, MA, USA), FOXO3 (1:1,000, Cell Signaling Technology, Beverly, MA, USA), ATG7 (1:1,000, Cell Signaling Technology, Beverly, MA, USA), and GAPDH (1:1,000, Santa Cruz Biotechnology, CA, USA). Membranes were subsequently incubated with HRP‐conjugated secondary antibodies for another 2 hr at room temperature. Immunoblots were visualized by an enhanced chemiluminescence detection kit (ECL kit; Millipore, Billerica, MA) under a chemiluminescence imaging analysis system (Amersham Imager 600, GE, CT, USA). Relative integrated density values were calculated using Image J software.

### 4.10 Cell transfection

The following plasmids were synthesized: short‐hairpin KCNQ1OT1 (sh‐KCNQ1OT1) plasmid and its respective nontargeting sequence (negative control, NC) (sh‐NC); FOXO3 full‐length (FOXO3 (+)) plasmid, short‐hairpin FOXO3 (FOXO3 (−)) and their respective nontargeting sequences (FOXO3 (+)‐NC or FOXO3 (−)‐NC); and short‐hairpin ATG7 (ATG7 (−)) and its respective nontargeting sequence (ATG7 (−)‐NC) (GenePharma, Shanghai, China). Cells were transfected as previously described. In brief, N2a cells were grown to 70%–80% confluency in 24‐well plates and then transfected with Lipofectamine 3,000 reagents (Invitrogen, CA, USA) according to the manufacturer's instructions. Stably transfected cells were selected using geneticin (G418; Sigma‐Aldrich, St. Louis, MO, USA). The efficiency of gene overexpression or knockdown was analyzed by qRT‐PCR. Four groups were designed to study the effect of KCNQ1OT1 on cells after OGD/R treatment: control, OGD/R, OGD/R + sh‐NC (transfected with empty plasmid), and OGD/R + sh‐KCNQ1OT1. To determine the role of FOXO3 in OGD/R, cells were divided into six groups: control, OGD/R, OGD/R + FOXO3(+)‐NC, OGD/R + FOXO3(+), OGD/R + FOXO3(−)‐NC, and OGD/R + FOXO3(−). To ensure the influence of ATG7 in OGD/R, cells were divided into four groups: control, OGD/R, OGD/R + ATG7(−)‐NC, and OGD/R + ATG7(−).

MiR‐200a agomir (pre‐miR‐200a), miR‐200a antagomir (anti‐miR‐200a), and their respective nontargeting sequences (negative control, NC) (pre‐NC or anti‐NC) were synthesized by GenePharma (Shanghai, China) to investigate the effect of miR‐200a in OGD/R. Cells were divided into six groups: control, OGD/R, OGD/R + pre‐NC, OGD/R + pre‐miR‐200a, OGD/R + anti‐NC, and OGD/R + anti‐miR‐200a. To confirm the hypothesis that KCNQ1OT1 functioned through suppressing miR‐200a, cells were divided into six groups: control, OGD/R, OGD/R + sh‐NC + pre‐NC (sh‐NC stable expressing cells co‐transfected with pre‐NC), OGD/R + sh‐KCNQ1OT1 + pre‐miR‐200a (sh‐KCNQ1OT1 stable expressing cells co‐transfected with pre‐miR‐200a), OGD/R + sh‐NC + anti‐NC (sh‐NC stable expressing cells co‐transfected with anti‐NC), and OGD/R + sh‐KCNQ1OT1 + anti‐miR‐200a (sh‐KCNQ1OT1 stable expressing cells co‐transfected with anti‐miR‐200a). To verify that miR‐200a might regulate autophagy via FOXO3, cells were divided into six groups: control, OGD/R, OGD/R + pre‐NC + FOXO3(+)‐NC (FOXO3(+)‐NC stable expressing cells co‐transfected with pre‐NC), OGD/R + pre‐miR‐200a + FOXO3(+)‐NC (FOXO3(+)‐NC stable expressing cells co‐transfected with pre‐miR‐200a), OGD/R + pre‐NC +FOXO3(+) (FOXO3(+) stable expressing cells co‐transfected with pre‐NC), and OGD/R + pre‐miR‐200a + FOXO3(+) (FOXO3(+) stable expressing cells co‐transfected with pre‐miR‐200a). Additionally, to explain that FOXO3 might affect cell viability through ATG7‐dependent autophagy, cells were divided into six groups: control, OGD/R, OGD/R + FOXO3(+)‐NC + ATG7(−)‐NC (FOXO3(+)‐NC stable expressing cells co‐transfected with ATG7(−)‐NC), OGD/R + FOXO3(+) + ATG7(−)‐NC (FOXO3(+) stable expressing cells co‐transfected with ATG7(−)‐NC), OGD/R + FOXO3(+)‐NC + ATG7(−) (FOXO3(+)‐NC stable expressing cells co‐transfected with ATG7(−)), and OGD/R + FOXO3(+) + ATG7(−) (FOXO3(+) stable expressing cells co‐transfected with ATG7(−)).

### 4.11 Cell viability assay

Cell viability was detected by Cell Counting Kit‐8 (CCK‐8, Dojindo, Japan) assay. N2a cells were seeded in 96‐well plates overnight at a density of 10^4^ cells per well. After OGD/R treatment, culture medium was added with 10 μl CCK‐8 solution in each well and incubated for 2 hr at 37°C. Absorbance was determined at 450 nm using a microplate reader (BioTek, Winooski, VT, USA).

### 4.12 Apoptosis analysis

Flow cytometry and terminal‐deoxynucleotidyl transferase‐mediated nick end labeling (TUNEL) assay were conducted to evaluate apoptosis. Cells were harvested and incubated in binding buffer with Annexin V‐PE and 7‐aminoactinomycin D (7‐AAD) (Becton Dickinson, CA, USA) according to the manufacturer's instructions. Samples were then analyzed using flow cytometry (FACSCalibur, Becton Dickinson) and CELL Quest software (Becton Dickinson). Cells with Annexin V+/7‐AAD‐ were identified as early apoptosis, while those with Annexin V+/7‐AAD+ as late apoptosis. Apoptotic morphology was visualized by TUNEL staining with the In Situ Cell Death Detection kit (Roche, Mannheim, Germany) in accordance with the manufacturer's protocol. The average number of TUNEL‐positive cells was calculated in five randomly selected fields.

### 4.13 Immunofluorescence staining

Cells were fixed with cold methanol at −20°C for 15 min and washed three times in phosphate‐buffered saline (PBS) for 5 min each. Cells were blocked in 5% normal goat serum for 1 hr at room temperature. The primary antibody against LC3B (1:100, Cell Signaling Technology, Beverly, MA, USA) was incubated at 4°C overnight. Cells were incubated with 1:100 TRITC‐conjugated anti‐rabbit IgG (Proteintech, Chicago, IL, USA) at room temperature for 2 hr. Nuclei were viewed with 4',6‐diamidino‐2‐phenylindole (DAPI) (Sigma‐Aldrich, St. Louis, MO, USA) after 5 min at room temperature. Cells were imaged using a laser scanning confocal microscope (Nikon, Japan).

### 4.14 Transmission electron microscopy

Cells were prefixed in 2.5% glutaraldehyde in 0.1 M phosphate buffer (pH 7.4) overnight and postfixed in 1% osmium tetroxide for another 2 hr at 4°C. After dehydration, infiltration, and imbedding, the samples were sectioned for TEM observation (Hitachi, Japan).

### 4.15 Autophagy flux detection

Adenovirus encoding mRFP‐GFP‐LC3 (Hanbio, Shanghai, China) was applied to infect cells. Cells were cultured for another 48 hr after infection and subjected to OGD/R. Cells were then fixed with 4% paraformaldehyde, and DAPI was used to show the nuclei. Imaging was performed by a laser scanning confocal microscope (Zeiss, Germany).

### 4.16 Mice miRNA microarrays

Microarray analysis was performed by Kangchen Bio‐tech (Shanghai, China) together with sample preparation and microarray hybridization.

### 4.17 Luciferase reporter assays

The supposed binding sites of miR‐200a with KCNQ1OT1 and the FOXO3 3′‐UTR fragment were cloned by PCR and inserted into a pmirGLO Dual‐luciferase miRNA Target Expression Vector (Promega, Madison, WI, USA) to create a luciferase reporter vector (KCNQ1OT1‐wt and FOXO3‐wt) (GenePharma). Corresponding mutants (KCNQ1OT1‐mut and FOXO3‐mut) (GenePharma) were constructed by gene alteration of the supposed binding sites. N2a cells were transfected with pmirGLO vectors and miRNA plasmids by Lipofectamine 3,000. Relative luciferase activities were evaluated using a Dual‐Luciferase Reporter Assay System (Promega) 48 hr after transfection.

Different promoter fragments of ATG7 were amplified from the genomic DNA, which was subcloned into a pGL3‐Basic‐Luciferase vector (Promega). pEX3 plasmids were constructed with full‐length mouse FOXO3 (GenePharma). The luciferase assay was conducted according to previous descriptions. The firefly luciferase activity was normalized by renilla luciferase activity.

### 4.18 Chromatin immunoprecipitation assay

ChIP assay was implemented using a Simple Chip Enzymatic Chromatin IP kit (Cell Signaling Technology) on the basis of manufacturer's protocols as previous described. Briefly, cells were suspended in 1% formaldehyde to cross‐link proteins to DNA and sonicated to average DNA fragment size. Lysates (2%) were applied as an input control. The other lysates were incubated with normal rabbit IgG or FOXO3 antibodies (Abcam, UK) for immunoprecipitation. DNA fragments were amplified by PCR. Primer sequences used for PCR are listed in Supporting Information Table S1.

## CONFLICT OF INTEREST

None declared.

## AUTHORS' CONTRIBUTIONS

SJY performed experiments, analyzed data, and wrote the manuscript; MJY analyzed bioinformatics and prepared the figures; XH, LLW, and ZQB performed statistical analyses; JF designed the experimental study and revised the manuscript. All authors approved the final version of the manuscript.
