# Supplementary figures and images for: KCNQ1OT1 promotes autophagy by regulating miR‐200a/FOXO3/ATG7 pathway in cerebral ischemic stroke
Source: Aging Cell. 2019 Apr 3;18(3):e12940. doi: 10.1111/acel.12940 (PMC6516167; doi:10.1111/acel.12940)

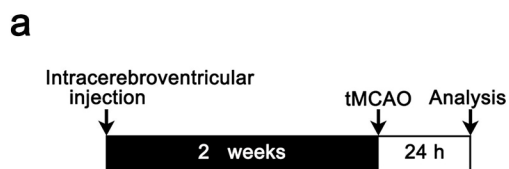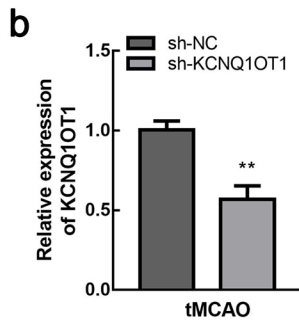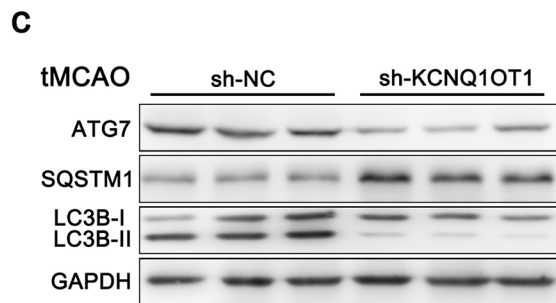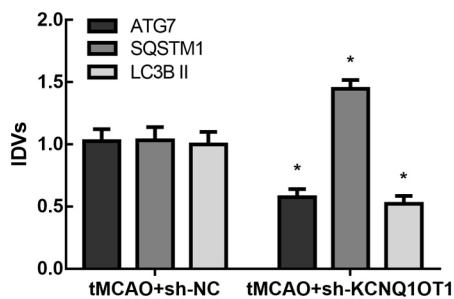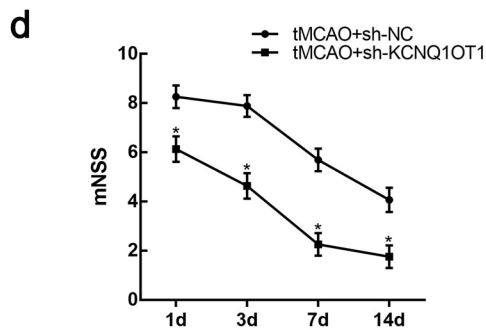

Fig S1

Supplement: Supplementary file 1 [file ACEL-18-e12940-s001.pdf]

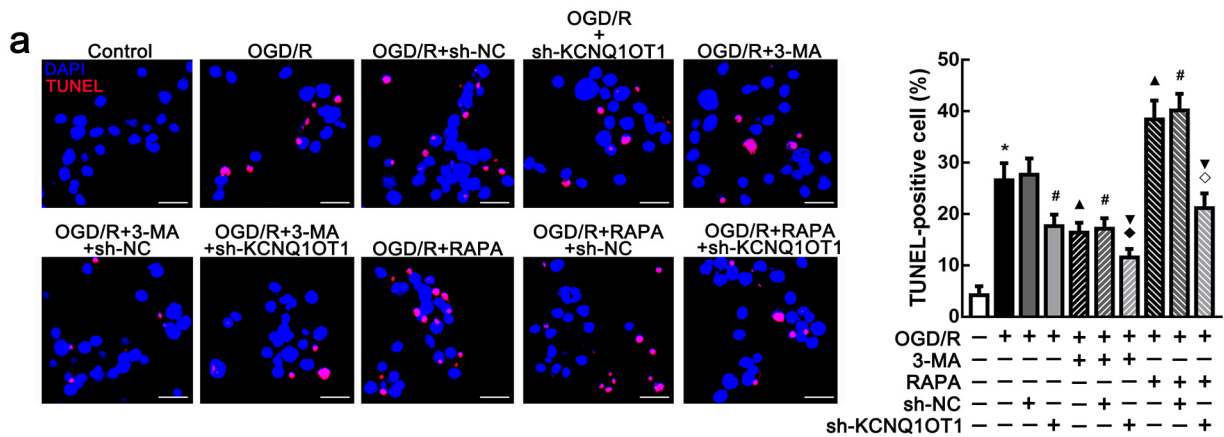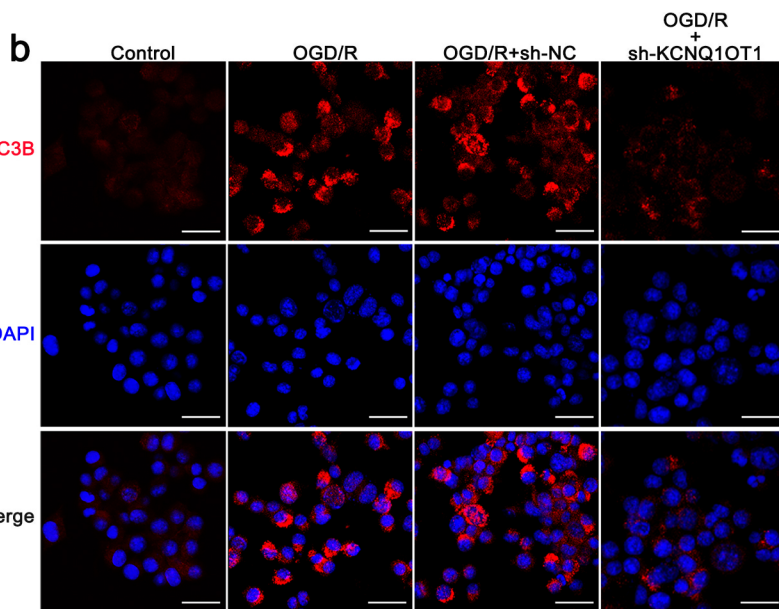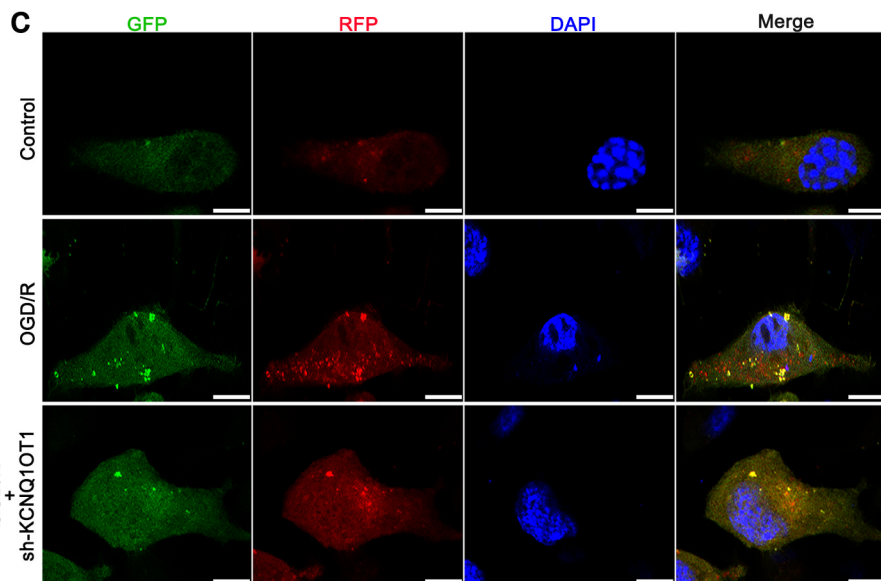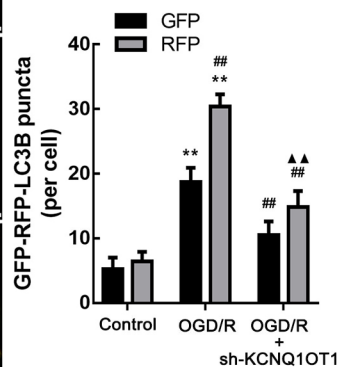

Fig S2

Supplement: Supplementary file 2 [file ACEL-18-e12940-s002.pdf]

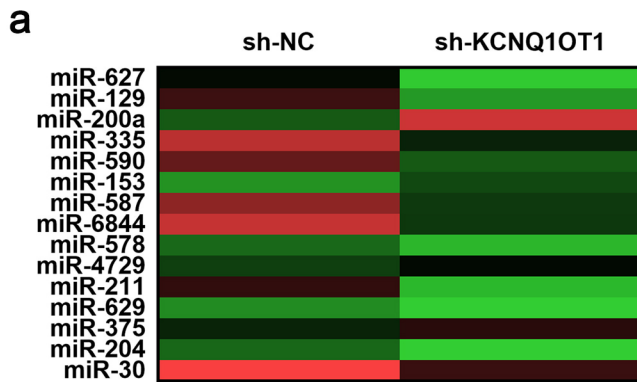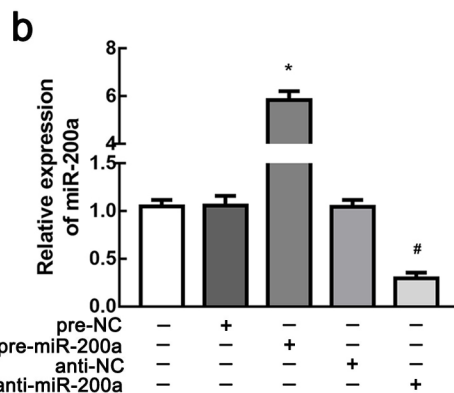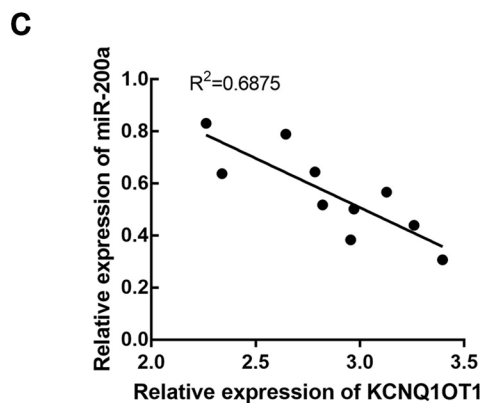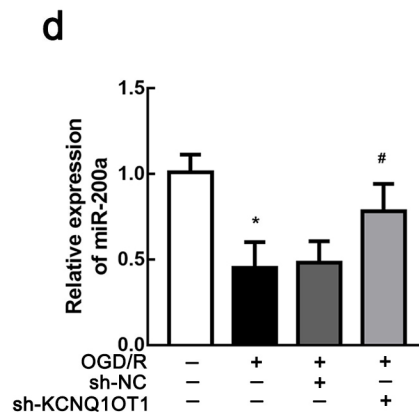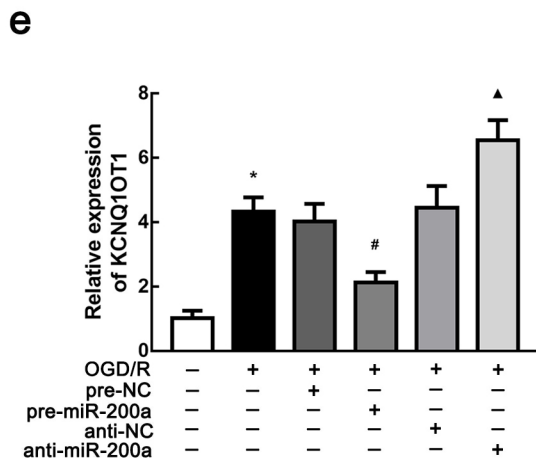

Fig S3

Supplement: Supplementary file 3 [file ACEL-18-e12940-s003.pdf]

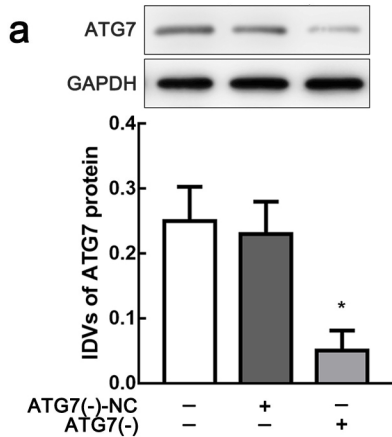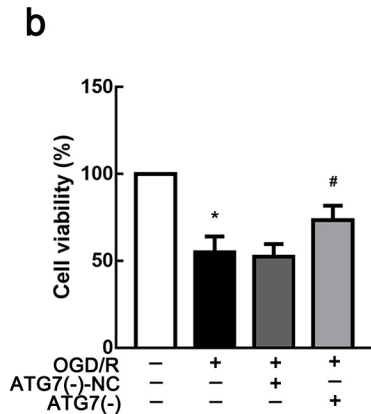

Fig S4

Supplement: Supplementary file 4 [file ACEL-18-e12940-s004.pdf]

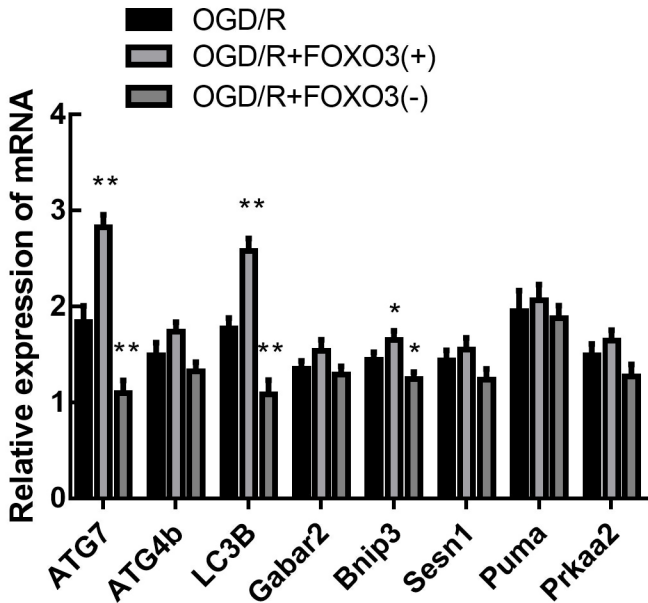

Fig S5

Supplement: Supplementary file 5 [file ACEL-18-e12940-s005.pdf]
