# Supplementary material for: KCNQ1OT1 promotes autophagy by regulating miR‐200a/FOXO3/ATG7 pathway in cerebral ischemic stroke
Source: Aging Cell. 2019 Apr 3;18(3):e12940. doi: 10.1111/acel.12940 (PMC6516167; doi:10.1111/acel.12940)
Supplement: Supplementary file 6 [file ACEL-18-e12940-s006.docx]

Table S1. Primers used for chromatin immunoprecipitation assay

| Control PCR1 |  |
| --- | --- |
| Forward primer | 5’-GGCCCAAAAGCCCTTTAACTTT-3’ |
| Reverse primer | 5’-TAACAACAAGAGCGATGACTGCTA-3’ |
| Product length | 167 |
| ATG7 PCR2 |  |
| Forward primer | 5’-ACCATGGTGATCATTCCTGTCA-3’ |
| Reverse primer | 5’- AACTTGAGTCGTGAGGAGGG-3’ |
| Product length | 175 |
| ATG7 PCR3 |  |
| Forward primer | 5’-GCCTTACAGGCCAGACAGAG-3’ |
| Reverse primer | 5’-GAAAAGGCCAGTGAACGTCG-3’ |
| Product length | 197 |
| ATG7 PCR4 |  |
| Forward primer | 5’-GATTACGTTCCTGGCCCAAAA-3’ |
| Reverse primer | 5’-CATAACAACAAGAGCGATGACTGC-3’ |
| Product length | 181 |
